# Hierarchical modeling of risk factors with and without prior information—the process of regression model evaluation for an example of respiratory diseases in piglet production from daily practice data

**DOI:** 10.3389/fvets.2025.1611771

**Published:** 2025-09-29

**Authors:** Timur Tug, Fiona Mers, Franziska Schäkel, Doris Höltig, Lothar Kreienbrock, Katja Ickstadt

**Affiliations:** ^1^Department of Statistics, TU Dortmund University, Dortmund, Germany; ^2^Institute of Biometry, Epidemiology and Information Processing, WHO Collaborating Centre for Research and Training for Health at the Human-Animal-Environment Interface, University of Veterinary Medicine, Hannover, Germany; ^3^Clinical Centre for Farm Animals, University of Veterinary Medicine, Hannover, Germany

**Keywords:** pork production, biosecurity, herd health management, respiratory health, hierarchical regression, frequentistic modeling, Bayesian modeling, model evaluation

## Abstract

In veterinary epidemiology, regression models are commonly used to describe animal health and related risk factors. However, model selection and evaluation present ongoing challenges—especially when many potential predictors, complex interactions, and limited sample sizes are involved. The VASIB project serves as a representative example, focusing on piglet-producing farms with persistent respiratory disease problems. Across 30 farms, a wide array of variables was collected at the farm, barn, compartment, pen, and individual animal levels, aiming to support optimized treatment and management strategies to improve respiratory health. This study investigates the occurrence of coughing in pigs using various epidemiological models, including hierarchical frequentist logistic regression, non-hierarchical Bayesian logistic regression (with full and partial pooling), and hierarchical Bayesian models with informative and non-informative priors. These approaches are evaluated and compared using statistical measures such as the corrected Akaike Information Criterion (AIC_c_), marginal and conditional R^2^, and intra-class correlation coefficients (ICC_c_/ICC_adj_). In the frequentist models, convergence issues arose due to limited observations within clusters, which did not occur in the Bayesian framework. While the choice of priors had limited influence on Bayesian model results, differences between models suggest that prior specification can still be relevant. Thus, it is important to assess and compare various model structures—including both hierarchical and non-hierarchical, and Bayesian versus frequentist approaches—to capture the data’s complexity and ensure robust inference. Here, the Bayesian hierarchical models outperform frequentist models, especially in handling complex data structures and providing robust estimates. Across all models, stocking density and floor condition emerged as consistently significant factors influencing the likelihood of coughing. Overall, this work emphasizes that there is no universal rule for model selection in veterinary data analysis. Instead, a balanced, context-sensitive modeling strategy that considers both statistical and epidemiological perspectives is essential to derive meaningful and actionable conclusions for improving animal health.

## 1 Introduction

The hygiene status of a farm is a major factor influencing animal health and is therefore a fundamental part of veterinary advice (1). However, the subjective rating of the veterinarian is prone to a lot of internal and external factors and may differ from visit to visit and can be difficult to understand. For this reason, careful evaluation is needed, which factors measure the health outcome on farms in a harmonized form and highlight critical points in an understandable way. In general, from the epidemiological point of view, this evaluation is in line with a careful development of regression models, connecting the health outcome with a more or less complex set of interacting factors under study, which describe the several biosecurity measures and other influencing factors (2–5).

In this work, the development of regression models for data from daily veterinary practice is described. Within the research project VASIB, in selected farms with sustainable respiratory disease problems, the aim is to examine whether targeted diagnostic measures, optimization of the treatment strategy and comprehensive, intensive management advice can minimize respiratory symptoms and with this the use of antibiotics and thus make an active contribution to reducing the general development of resistance in livestock farming. To this end, the project is working on the development and validation of a model that can be used with onsite-farming data from veterinary practices with the aim of synergizing epidemiological data from veterinary preventive medicine and farm data (6).

However, to describe respiratory health in piglet production different herd measures, which are based on direct veterinary inspection and on information from the farmers may be used. Overall, this may be interpreted as a multivariate health outcome or the need of selection a representative surrogate to describe respiratory health. Within this investigation we choose as a surrogate “coughing in piglets,” which is generally used in practice (7).

Nevertheless, many other factors must be considered, if coughing has to be described during the veterinary inspection visit. First, animal health data on a farm appears at farm, barn, compartment, pen and individual animal level. These hierarchical structures must be considered especially if factors respond in different ways, like the air- or feed borne transmission (8). Second, manyfold direct (causal) and indirect factors effect animal health, which are more or less associated within an interacting and partial correlated structure (9, 10). And, if these multiple factors lead to a large number of different classes, they break down into multiple substructures, which usually do not contain a sufficient number of animals for a powerful epidemiological analysis. This at the end, causes missing data, which finally restricts the prognostic value of an epidemiological model (3).

Against this background the development of an epidemiological statistical model to be used for prevention in livestock farming is not a matter of highest quality and precision only, but a sophisticated model building process, which takes into account the needs of daily work data.

Classical statistical models often struggle to account for complex data structures, leading to potential bias when questionable or poorly defined covariates are included (3). To address this, advanced statistical methodologies, such as hierarchical or generalized models, are employed to better capture variability and dependencies in the data (4). Bayesian methods are particularly valuable in cases where prior knowledge exists, enabling the incorporation of expert insights into the modeling process for more robust and informed inference (11).

The objective of this paper therefore was twofold: first, to evaluate and compare the performance of different hierarchical regression modeling approaches—both frequentist and Bayesian—applied to complex, nested data from piglet production systems, and second, to investigate the relevance of selected environmental and management-related predictors, such as floor condition and stocking density, on coughing as a clinically meaningful indicator of respiratory health in weaner pigs. This modeling process presented here is intended to support veterinary decision-making not only within the VASIB project, but also in routine farm settings.

## 2 Material and methods

### 2.1 Study design and data acquisition

The data used for this investigation was enrolled during the VASIB project on farms that were supervised by one veterinary practice network that is located in the Federal States of North Rhine Westphalia and Schleswig Holstein, Germany. Only farms with piglet and weaner production with sustainable respiratory health problems were selected for this investigation. Within this setting this study followed a single cross-sectional design. Data collection took place between April and November 2016 during scheduled veterinary visits.

For this paper, the primary health issue addressed is coughing, which in daily routine practice serves as a symptom indicative of underlying respiratory diseases in weaner pigs. The assessment focuses on multiple hygiene and management measures that can influence the respiratory health of pigs. Therefore, the basic health problems and biosecurity measures included in the assessment are:

1. Coughing: the primary indicator of respiratory distress in pigs, often linked to various environmental and management factors.
2. Barn hygiene: this includes cleanliness and the presence of contaminants in the barn environment, which can exacerbate respiratory issues.
3. Disposal hygiene: the management of carcasses and waste, which if poorly handled, can lead to health risks for living animals.
4. Isolation and transport: the conditions under which pigs are transported and isolated, impacting their exposure to stress and pathogens.
5. Hygiene of drug administration: the protocol for administering medications can affect the overall health and recovery of the animals.
6. Feed hygiene: the quality and cleanliness of feed can influence the health status of the pigs and their susceptibility to diseases.
7. Climate control: proper ventilation and climate conditions in barns are critical to maintaining respiratory health and preventing disease outbreaks.
8. Management husbandry: overall management practices, including grouping and caring for the animals, which can influence their health status.
9. Cleaning and disinfection: the frequency and effectiveness of cleaning procedures can prevent disease spread and improve overall hygiene in the barn environments.

For this, 30 piglet-producing farms with recurring respiratory tract disease problems in weaners were selected for an in-deep investigation. Overall, data was enrolled within 72 pigsties, 130 compartments, and 300 pens respectively, and finally from 450 single animals. Although all farms were part of a common veterinary network, they were managed independently and did not belong to the same corporate ownership. Thus, differences in management style, resource availability, and biosecurity adherence exist. To partially control for such unobserved heterogeneity, we used hierarchical modeling with random effects at the compartment and pen levels, which helps account for unmeasured clustering effects. However, we acknowledge that certain latent factors at the farm level—such as corporate protocols or feeding systems—were not directly modeled and may introduce unmeasured confounding. This limitation is discussed later in the discussion Section.

In preparation for the veterinary visit, a questionnaire was developed and evaluated beforehand. The questionnaire included management, biosecurity, feeding, medication and medical history aspects (181 items: 81% closed, 7% semi-closed, 12% open questions) was sent to the farmers. This information was verified and completed during a face-to-face interview at the start of the farm visit and missing values, or implausible answers were clarified (the original German version is on view at: https://www.tiho-hannover.de/institut-fuer-biometrie-epidemiologie-und-informationsverarbeitung/publikationen/zusatzmaterial-publikationen).

The information of the questionnaires, the checklist and the results of the clinical examination were entered in a SQL database developed for the study. All datasets were checked for plausibility and completeness.

For demonstration of the regression modeling process, the variables addressed in Table 1 were selected for this investigation.

**Table 1:** Variables and descriptions in the “initial” dataset.

Variable	Description (categories)	Level
Cough	Do the pigs cough? (0 = no; 1 = yes)	Animal
Clinical variables	Sum of all clinical variables (0 = no symptoms; 1 = mild symptoms)	Animal
Laboratory variables	Sum of all laboratory variables, including blood	Animal
Blood lab variables	Sum of blood lab variables	Animal
Pen ID	ID variable for the pens	Pen
Age	Age of animals (in days)	Pen
Animal contamination	Degree of dirtiness of the animals (0 = no findings; 1 = slightly dirty; 2 = moderately dirty; 3 = heavily dirty)	Pen
Skin injuries	Animals with skin injuries (0 = none; 1 = few (up to 10%); 2 = some (up to 50%); 3 = many (over 50%))	Pen
Pen size	Size of the pen (in m^2^)	Pen
Stocking density	Stocking density in the pens	Pen
Compartment ID	ID variable for the compartments	Compartment
Water flow rate	Water flow rate (in ml/min)	Compartment
Temperature	Recorded temperature (in °C)	Compartment
Air pressure	Air pressure (in Pa)	Compartment
CO_2_ level	CO_2_ level (in ppm)	Compartment
Relative humidity	Relative humidity (in %)	Compartment
NH_3_ level	NH_3_-adjusted value (in ppm)	Compartment
Floor condition	Condition of the floor (1 = new; 2 = moderately worn; 3 = heavily worn; 4 = damaged)	Compartment
Farm ID	ID variable for the farms	Farm
Disinfectant	Frequency of disinfectant replacement in disinfection baths (1 = daily; 2 = weekly; 3 = when dirty; 4 = after emptying; 5 = irregularly)	Farm
Proximity to next farm	Proximity to the nearest pig farm (1 = <0.5 km; 2 = 0.5 km – 10 km; 3 = > 10 km)	Farm
Target temperature – In	Average target temperature when animals are housed (in °C)	Farm
Target temperature – Out	Average target temperature when animals are removed (in °C)	Farm
Respiratory diseases	Batches affected by respiratory diseases last year (1 = few (up to 10%); 2 = some (up to 50%); 3 = many (over 50%))	Farm
Protective clothing	Use of protective clothing outside barns (0 = no; 1 = yes, to cross the yard; 2 = yes, for other tasks)	Farm
Minimum quarantine	Minimum quarantine duration (in days)	Farm
Maximum quarantine	Maximum quarantine duration (in days)	Farm
Winter	Was the farm visited in winter? (0 = no; 1 = yes)	Farm

**Figure 1:**
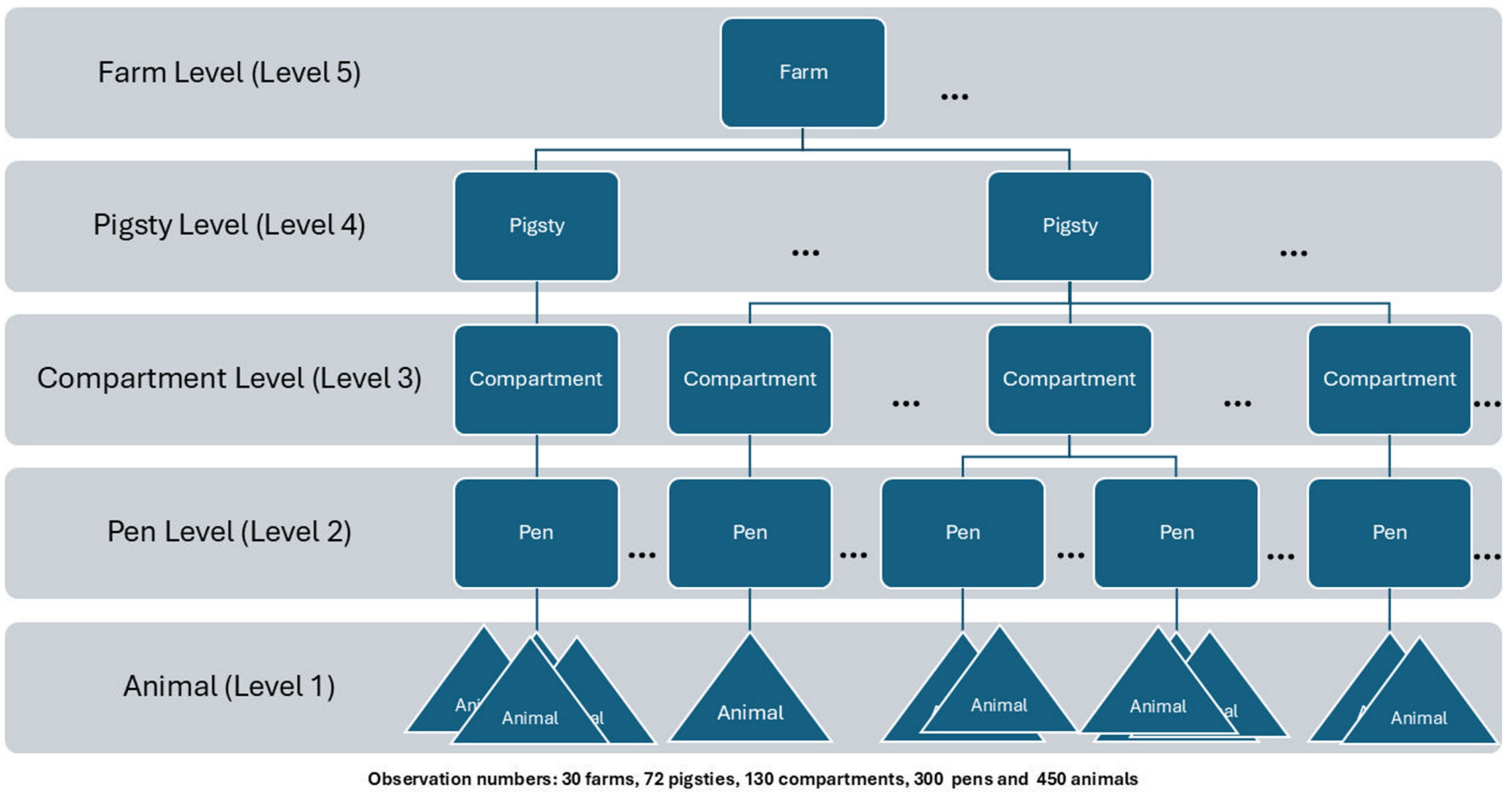
Hierarchical levels within the sample population.

In order to investigate the influence on cough in the study population hierarchical logistic (frequentist and Bayesian) regression models were used. Here, we include all levels [farms (30) → pigsties (72) → compartments (130) → pens (300)] as mentioned above. The pen sizes vary between 12 and 85 animals, from which overall 450 animals were included into individual veterinary inspection (see Figure 1). The selection of animals was based on practical feasibility and clinical judgement during the veterinary inspection. Animals showing respiratory signs, especially coughing, were prioritized for inclusion. However, non-coughing animals were enrolled as well.

For these analyses, Table 1 presents information derived from 1,298 variables, encompassing both qualitative and quantitative data. To reduce this number of variables to Table 1, an initial screening was conducted, during which all items were inspected for their relevance. Missing values were present in several variables, primarily due to incomplete documentation during routine on-farm assessments and occasional technical measurement issues. The overall proportion of missing values across the dataset was 23.1%, with individual variables showing between 0 and 100% missingness. Variables with more than 50% missing values (a total of 136 variables) were excluded, along with alphanumeric variables (45 variables) such as comments and text fields. Additionally, categorical factor variables with only a single level of expression (121 variables) were eliminated due to insufficient variability. Following this data cleaning, 228 variables remained in this dataset. To address potential multicollinearity, a heterogeneous correlation matrix, consisting of the usual Pearson product–moment correlation for continuous variables, the biserial or polyserial correlation for mixed continuous categorical variables and the polychoric correlations between several multilevel categorical variables (38, 39, p. 100f.) was computed. Highly correlated variables (correlation coefficient > 0.8) were removed. Following preprocessing, 29 variables, included both qualitative (categorical) and quantitative (continuous) measures, remain for further analysis.

Approximately 15% of the data in the first-visit dataset were missing and were handled using multiple imputation to ensure data integrity and avoid bias in the results. For quantitative variables, the predictive mean matching (PMM) method was used, in which real values are drawn from the available data to generate plausible replacement values for missing entries (12). Categorical variables (see Section 3.1 and Table 1) were imputed using a proportional odds logistic regression (POLR) model to adequately account for ordinal relationships between categories (13, 14). These methods made it possible to minimize the impact of missing data and perform a more robust analysis of factors influencing cough in pigs. The R software packages “mice” and “miceadds” are used for both imputation methods (15, 16). The hierarchical structure of the data—comprising animals nested within pens, compartments, and farms—was explicitly taken into account to ensure that the imputation procedure met the specific requirements of the dataset (13, 14). In order to address this structure, group-level identifiers (pen ID, compartment ID, farm ID) were included as auxiliary variables in the imputation models. Incorporating these identifiers as predictors enabled the procedure to capture clustering effects and to reflect the multilevel dependencies inherent in the data. By doing so, the imputation models preserved intra-cluster correlations and increased the plausibility of the imputed values under the assumption that data were missing at random (MAR).

The outcome variable, “coughing in piglets,” was not formally tested for conditional independence prior to model fitting. Conditional independence implies that the association between two variables disappears when conditioning on a third variable. While such tests can be informative, especially in causal modeling frameworks, they are often difficult to implement and interpret in the presence of multiple covariates and hierarchical data structures. Given the complexity and high dimensionality of the dataset, we prioritized the specification of hierarchical models that account for confounding and clustering effects by design, rather than performing separate conditional independence tests. Additionally, predictor selection was based on theoretical knowledge and previous studies.

Assumptions regarding independence, particularly in relation to modeling components such as Gaussian priors in the Bayesian framework, should be interpreted cautiously. However, the hierarchical data structure and relevant dependencies were explicitly addressed through appropriate model specification using both frequentist and Bayesian approaches.

### 2.2 Classical analysis of hierarchical model

Hierarchical regression extends classical regression by handling clustered data structured across multiple levels (see Figure 1). Here, the goal is to account for the variability at each level to analyze cluster effects (5). The key elements are varying coefficients and a model for these coefficients, possibly incorporating cluster-level predictors—features that distinguish hierarchical models from classical ones. Here, we examine frequentist hierarchical models and compare their applicability in the context of cough prevention in pig production.

For multilevel data, each level contributes a variance component that measures intraclass-correlation. As an example, we here consider a three-level model for the cough response, $y_{\mathrm{ijk}}\sim \mathrm{Ber}(\pi_{\mathrm{ijk}}),$ with cough probability $\pi_{\mathrm{ijk}}$ for the k -th pig, located in the j -th pen in the i -th compartment. Pigs (level 1) are nested in pens (level 2), which are nested in compartments (level 3); compartments are the primary units, pens the secondary units, and pigs the units of observation. These clusters are treated as random effects with an average effect of zero, and the analysis is performed using logistic regression. In addition to random effects capturing the hierarchical structure (e.g., pens, compartments), relevant predictors at the pig, pen and compartment levels—such as animal age, pen size, stocking density, environmental conditions, and hygiene indicators—are included as fixed effects in the hierarchical regression models. With this, the data are modeled by a logistic regression model (see Equation 1).


(1) $$\log(\frac{\pi_{\mathrm{ijk}}}{1-\pi_{\mathrm{ijk}}})=\beta_{0\mathrm{ij}}+\beta_{1}\mathrm{pi}g_{\mathrm{ijk}}^{1}$$

where $\beta_{0\mathrm{ij}}$ is the intercept, $\beta_{1}$ the coefficient associated with the pig-level predictor $\mathrm{pi}g_{\mathrm{ijk}}^{1}$ for $k=1,\ldots ,n_{\mathrm{ij}}$ pigs, $j=1,\ldots ,n_{i}$ pens and i=1,…,n,(n∈ℕ) compartments.

Each pen is modeled with its own logistic regression. The pig-level predictor $\mathrm{pi}g^{1}$ has the same effect across pens, while the pen-specific *β*₀ᵢⱼ captures differences between pens (see Equation 2):


(2) $$\beta_{0\mathrm{ij}}=\beta_{0i}+\beta_{2}\;\mathrm{pe}n_{\mathrm{ij}}^{2}+u_{0\mathrm{ij}}$$

where $\mathrm{pe}n_{\mathrm{ij}}^{2}$ is a pen-level covariate and $u_{0\mathrm{ij}}$ a pen-level random effect. At the compartment level, the intercept is modeled as


(3) $$\beta_{0i}=\beta_{0}+u_{0i}$$

Substituting (2) and (3) into (1) yields


(4) $$\begin{array}{l} \log(\frac{\pi_{\mathrm{ijk}}}{1-\pi_{\mathrm{ijk}}})=\beta_{0}+\beta_{1}\mathrm{pi}g_{\mathrm{ijk}}^{1}+\beta_{2}\mathrm{pe}n_{\mathrm{ij}}^{2}+\\ \;\beta_{3}\text{compartmen}t_{i}^{3}+u_{0i}+u_{0\mathrm{ij}}. \end{array}$$

Here, $\beta_{3}$ represents an additional compartment-level covariate included as a fixed effect. This generalized linear mixed model includes two random effects— $u_{0i}\;$ for compartments ( $u_{0i}\sim N(0,\sigma_{0i}^{2})$ ) and $u_{0\mathrm{ij}}$ for pens ( $u_{0\mathrm{ij}}\sim N(0,\sigma_{0\mathrm{ij}}^{2}))$ —which account for cluster-specific variability (5). In practice, as in our final multivariable model fitted in R (see Section Results), multiple predictors at different levels were included simultaneously (e.g., animal age, pen size, stocking density, CO₂ concentration, ammonia levels, and relative humidity). This reflects the actual complex production setting more accurately than the simplified didactic formulation in Equations 1–4.

As illustrated in the VASIB example, with only five individuals per cluster, variance estimates may be unreliable, and substantial variability in the random effects can lead to biased estimates of the fixed effects. Hierarchical models are thus better able to handle dependencies in nested data than non-hierarchical approaches (17). Estimation of variance components is unreliable with as few as five individuals per cluster (17), and high variability in random effects can bias estimates, making hierarchical models that incorporate random effects to account for nested data superior in accuracy and fit to non-hierarchical models that assume independent observations.

### 2.3 Bayesian analysis of hierarchical model

Bayesian regression estimates parameters as distributions by combining sample data with prior knowledge, making it useful for complex relationships, non-convergence in maximum likelihood (ML) methods, and small samples (18). Non-hierarchical Bayesian models assume independence and ignore clustering, thereby risking bias, while hierarchical Bayesian models assign priors to capture the nested data structure.

For example, parameters such as $\beta_{0}$ (intercept) and $\beta_{1}$ (pig-level effect) can be modeled with Gaussian priors, $\beta_{0},\beta_{1}\sim N(\mu ,\sigma^{2})\;$ treating the data as a single population (“complete pooling”). The response variable is modeled as $y_{\mathrm{ijk}}|\pi_{\mathrm{ijk}},\theta \sim \mathrm{Ber}(\pi_{\mathrm{ijk}})$ with prior vector *θ* containing all level-specific *β* coefficients. For the compartment-specific intercepts, we assume (5)–(7):


(5) $$\beta_{0i}|\beta_{0},\sigma_{0}\sim N(\beta_{0},\sigma_{0}^{2})$$

(6) $$\beta_{0}\sim N(\mu ,\sigma^{2})$$

(7) $$\sigma_{0}\sim \text{InvGamma}(a,b)$$

The level-specific regression coefficients follow (8):


(8) $$\beta_{1},\ldots ,\beta_{p}\sim N(\mu ,\sigma^{2})$$

Continuous predictors are standardized before model fitting, and parameter estimates are later back transformed to the original scale for interpretation. Specifically, the intercept on the original scale is obtained as


(9) $${\tilde{\beta }}_{0}={\hat{\beta }}_{0}-\sum_{m=1}^{p}{\hat{\beta }}_{m}\frac{{\bar{x}}_{m}}{S_{m}}$$

where ${\bar{x}}_{m}$ and *Sm* are the mean and standard deviation of the *m*-th predictor, respectively. The regression coefficient of the *m*-th predictor on the original scale is then,


(10) $${\tilde{\beta }}_{m}=\frac{{\hat{\beta }}_{m}}{S_{m}}$$

Overall, the full Bayesian hierarchical model with highly informative priors is specified as (11):


$$\begin{array}{l} \log(\frac{\pi_{\mathrm{ijk}}}{1-\pi_{\mathrm{ijk}}})=\beta_{0}+\beta_{1}\mathrm{pi}g_{\mathrm{ijk}}^{1}+\beta_{2}\mathrm{pe}n_{\mathrm{ij}}^{2}+\\ \;\beta_{3}\text{compartmen}t_{i}^{3}+u_{0i}+u_{0\mathrm{ij}}, \end{array}$$

with random effects


$$u_{0i}\sim N(0,\sigma_{0i}^{2})\;\text{and}\;u_{0\mathrm{ij}}\sim N(0,\sigma_{0\mathrm{ij}}^{2}),$$

and priors


$$\beta_{0}\sim N(0,1)$$

$$\beta_{1},\ldots ,\beta_{15}\sim N(0,1)$$

$$\sigma_{0i}\sim \text{InvGamma}(0.5,0.5)$$

(11) $$\sigma_{0\mathrm{ij}}\sim \text{InvGamma}(0.5,0.5)$$

In practice, as in our final multivariable Bayesian model fitted in R (see Section Results), multiple predictors at different levels were included simultaneously (e.g., animal age, pen size, stocking density, CO₂ concentration, ammonia levels, and relative humidity). This reflects the actual complex production setting more accurately than the simplified didactic formulation in Equations 5–11. Unlike the frequentist models, Bayesian inference yields full posterior distributions for each parameter, allowing results to be summarized by means and 95% credible intervals rather than point estimates with confidence intervals.

In this investigation, we compare the final multivariable frequentist hierarchical model (FM 1–3) with three Bayesian logistic regression models: a non-hierarchical model with non-informative priors (BM 1), a hierarchical model with non-informative priors (BM 2 and 3), and a hierarchical model with highly informative priors (BM 4 and 5). While additional frequentist models were used during model development (e.g., univariable screening), only the final model (FM 2) was used for direct comparison with the Bayesian models.

Firstly, it should be noted that we employ Markov Chain Monte Carlo (MCMC) algorithms for model fitting. For all Bayesian models, four independent chains were run with 5,000 iterations each, of which the first 2,500 iterations per chain were used for warm-up. This leaves us with a total of 10,000 post-warmup draws. Calculations are done with the brms R package, version 2.17.0, and the statistical software R, version 4.0.5 (19). The brms package (20–22), with the help of the rstan package (23), uses the Stan platform to fit Bayesian hierarchical models. For further calculations and graphical representation ggmcmc (24), ggplot2 (25), bayesplot (26, 27), performance (28), tidybayes (29) and lme4 (30) were used.

### 2.4 Evaluation measures

In this study, several goodness-of-fit measures were employed to assess and compare model performance in both Bayesian and/or frequentist frameworks. Two key metrics were the R^2^ measures and the Akaike Information Criterion (AIC), along with its corrected version (AIC_C_), as well as the Intraclass-Correlation Coefficient (ICC).

The R^2^ measure is divided into two types for hierarchical models: the marginal R^2^ (R^2^_m_) and the conditional R^2^ (R^2^_c_) (31). The marginal R^2^ represents the variance explained solely by the fixed effects, while the conditional R^2^ accounts for the total variance explained by both fixed and random effects. A large difference between these two indicates that a substantial portion of the variance is attributable to the grouping (random) effects, emphasizing the importance of properly modeling the hierarchical structure. In addition to the other model validation metrics, we calculated the Bayesian R-squared (Bayes R^2^) to assess the proportion of variance explained by the model. Unlike classical R^2^, the Bayesian R^2^ is derived from the posterior predictive distribution, providing a distribution of R^2^ values rather than a single point estimate. To summarize this distribution, we report the posterior median of Bayes R^2^ along with a 95% credible interval, which reflects the uncertainty inherent in the model fit. This approach allows us to present a single, interpretable R^2^ value while acknowledging the variability in model performance due to posterior uncertainty.

The ICC further breaks down the variance by measuring the proportion attributable to the random grouping factors (such as compartments or pens). This measure is critical for hierarchical models as it quantifies the degree of similarity within clusters. An adjusted ICC, which considers only the variance of the random effects relative to the total variance (random effects plus residual error), provides insight into the cluster-specific influence on the outcome. In the Bayesian models, a single intra-class correlation coefficient (ICC) value was calculated based on the posterior distributions of the random effects. Specifically, the ICC was derived as the median of the posterior samples for the ratio of the random effect variance to the total variance (sum of the random effect variance and residual variance). This approach allows for a representative point estimate from the posterior distribution and follows the general recommendation for hierarchical models as outlined by Gelman and Hill (4). For the evaluation of the Bayesian models, Leave-One-Out Cross-Validation (LOO-CV) was employed to assess predictive performance. The LOO approach was implemented using the loo package in R, which provides an efficient approximation of out-of-sample prediction error based on the expected log pointwise predictive density (elpd). Detailed results and comparisons between models using LOO are presented later in Section 3.4.

In frequentist models, these measures are derived from maximum likelihood estimates and are used alongside the AIC and AIC_C_ to compare models. The AIC balances model fit against complexity, where lower values indicate a better trade-off between the goodness of fit and parsimony. The AIC_C_ further adjusts for small sample sizes, providing a more reliable basis for model.

## 3 Results

### 3.1 General data structure and description of the sample population

After the data cleaning and imputation processes, the “initial” dataset for this paper contained a total of 29 variables (see Table 1), whereby these variables are both qualitative and quantitative in nature. The basis descriptive measures of these variables are displayed in Table 2. A critical assessment revealed that all variables associated with the pen level of the animals were highly correlated. This high degree of correlation indicated low variability between the pens within each farm. Specifically, in 27 out of the total 30 farms, all pigs were sourced from a single pen, underscoring the lack of diversity in pen conditions. Consequently, it was decided to exclude the pen level from further analyses to enhance the robustness of the results and reduce the risk of unstable parameter estimates due to multicollinearity.

**Table 2:** General descriptive measures of the sample population (*n* = 450 animals from 30 farms).

Quantitative variables				
Variable	Mean	Std. dev	Min	Max
Age in days	52.067	15.356	28.000	89.000
Pen size in m^2^	12.223	4.715	5.760	25.750
Stocking density in animals/m^2^	0.388	0.157	0.170	1.080
Water flow rate in ml/min	999.422	477.069	100.000	2,200.000
Temperature in °C	27.767	1.938	22.000	32.800
Air pressure in Pa	1,009,750	10.101	989.000	1,032.200
CO_2_ in ppm	2,247.556	980.917	800.000	5,000.000
NH_3_ in ppm	9.073	6.312	2.000	30.150
Relative humidity in %	63.748	7.963	45.200	78.500

Qualitative variables			
Variable	Category	*n*	%
Floor condition	Moderately worn	308	68.4
	New	142	31.6
Skin injuries 1	No	259	57.6
	Yes	191	42.4
Skin injuries 2	No	346	76.9
	Yes	104	23.1
Animal contamination 1	No	217	48.2
	Yes	233	51.8
Animal contamination 2	No	310	68.9
	Yes	140	31.1
Cough	No	264	58.7
	Yes	186	41.3

As described in Section 2, the dataset comprised 30 farms with 15 observations each (total *n = 450*), after correlated variables had been filtered out. Thus, the models were fitted to a streamlined and meaningful set of predictors relevant to the study objectives. The most important 14 variables are described descriptively in the following Table 2. Density plots of observed vs. imputed values for selected numerical variables, as well as convergence diagnostics (mean and standard deviation over 20 iterations), are provided in the Supplementary Figures S1–S3. Density plots were not generated for categorical variables, as they are not appropriate. The displayed trends indicate normal variation and convergence, with target values summarized in Table 2.

A total of 450 animals were included in this study. The average age of the animals was approx. 52 days, with an age range of 28 to 89 days. The average pen size was approximately 12 m^2^, with a range of 5.760 to 25.750 m^2^. The stocking density averaged 0.388 animals per m^2^, i.e., 1 animal per 2.577 m^2^. Floor conditions were predominantly classified as moderately worn (68.4%), while only a small proportion of pens were classified as new (31.6%). The water flow rate averaged 999 mL/min, with a range from 100 to maximum values of up to 2,200 mL/min. The average temperature was a pleasant 27.8 °C. The mean air pressure was approximately 1,009.8 Pa, with a range from a minimum of 989 Pa to a maximum of 1,032 Pa. The CO_2_ value averaged about 2,248 ppm and ranged from 800 ppm to 5,000 ppm; while the NH_3_ value averaged about 9.1 ppm and varied between 2 ppm and 30 ppm. The relative humidity of approximately 63.8%, which varied between 45.2 and 78.5%.

Finally, 264 animals reported no coughing (58.7%), while 186 animals suffered from coughing (41.3%). For the analyses, we standardized the numerical variables and used them for the calculation. These results provide valuable insights into the health and husbandry conditions of the animal populations studied and their potential impact on animal welfare.

### 3.2 Results of frequentist models

Starting the model selection process, we fit three hierarchical frequentist models (FM) without any explanatory variables first to assess the impact of the cluster structure of the data. Table 3 shows the basic characteristics and measures of model fit for these basic models.

**Table 3:** Measures for model specificity comparing hierarchical frequentist model (FM) 1, 2 and 3.

	FM 1	FM 2	FM 3
Model with random intercepts (for each)	Pens	Pens, compartment	Pens, compartment, farms
Estimated intercept	−0.62	−0.61	−0.58
Log-likelihood	−289.63	−270.97	−270.04
Estimated variance pens	2.29	0.20	0.22
Estimated variance compartments		2.84	1.96
Estimated variance farms			0.90
R*_c_*^2^	0.36	0.43	0.43
ICC*_adj_*	0.41	0.48	0.48

From the model estimates (using the Laplace approximation; see Table 3 for details) the log-likelihood of a pig coughing in an “average” pen is estimated as $\hat{\beta_{0}}=-0.62$ , i.e., the probability of suffering from coughing without the influence of other variables is $\frac{\exp(-0.62)}{1+\exp(-0.62)}\;$ =0.35 or 35%. The adjusted intraclass-correlation ( $\mathrm{IC}C_{\mathrm{adj}}$ ) shows that between 41 and 48%, respectively, of the variation (compared with the total variance) in the outcome variable cough can be explained by the respective clustering structure of the data in the models. For FM 2 and FM 3, the ICC-values are the same, as adding the farm level does not seem to provide any additional information. There is not enough additional farm-level variation to justify adding an additional random effect at this level to explain all of the observed variation. Although the FM 1 confirms that there is variation between pens, the magnitude of this variation can be nearly fully explained by the variation between compartments and the residual variance term. It should be noted that the FM 3 failed to converge, even though we have yet to add explanatory variables to the models. Therefore, the farm level was not further investigated in the model building process.

**Figure 2:**
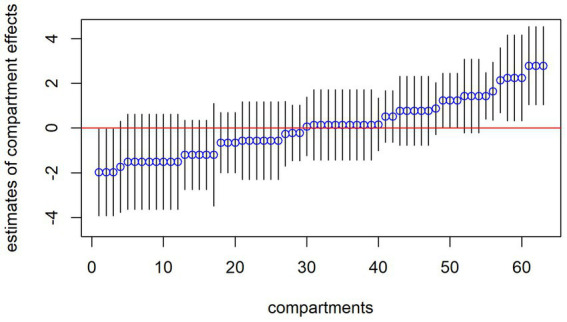
Confidence intervals of estimated residuals per compartment from FM 2 with circles showing modes (intercept-only).

Before including explanatory variables in the model, the estimates of the compartmental effects or residuals, $\hat{u_{0i}}$ have to be considered in more detail. These are calculated from the FM 2. Figure 2 plots these conditional modes of the random compartment effect with all 63 compartments in total in rank order along with the associated 95%-confidence intervals. The graph shows the estimated residuals for all compartments in the sample after remaining preprocessing. For nine of the 63 total compartments, the 95%-confidence intervals do not overlap with the horizontal line at zero, indicating that coughing in these compartments is above average. The confidence intervals are quite wide for some compartments, which is in line with the restricted sample sizes within these compartments. A corresponding graph (for FM 2) of the pen effects would simply consist of a horizontal line at zero, so this is not shown here.

Subsequently, explanatory variables are included in the model in addition to the random intercepts. The starting point is initially the FM 2 from above. For model 1 the variables collected at pen level are included first. These are the age of pigs in a specific pen, the pen size or the stocking density in a pen and others. Model 1 assumes that the relationship of the explanatory variables with cough is the same across pens or compartments. Furthermore, we now add explanatory variables collected at the compartment level to our model. For both models, a random intercept is allowed for each of the pens or compartments. Table 4 shows the resulting odds ratios with associated 95%-confidence intervals for the fixed effects of the fitted hierarchical logistic regression models (FM 2), where model 1 has explanatory variables for pen level and model 2 has explanatory variables for the pen- and compartment level.

**Table 4:** Odds ratios and 95% confidence intervals for fixed effects in frequentist hierarchical logistic regression models.

Factor	Model 1	Model 2
Age (in days)	**1.70 [1.19, 2.42]**	**1.64 [1.13, 2.38]**
Pen size (in m^2^)	0.71 [0.47, 1.06]	0.94 [0.60, 1.48]
Stocking density (in animals/m^2^)	1.26 [0.93, 1.70]	1.27 [0.92, 1.75]
Animal contamination 1 (none vs. low)	1.37 [0.63, 2.99]	1.05 [0.46, 2.40]
Animal contamination 2 (none vs. high)	1.06 [0.39, 2.85]	0.63 [0.21, 1.88]
Skin lesions 1 (none vs. low)	0.86 [0.45, 1.64]	1.02 [0.51, 2.05]
Skin lesions 2 (none vs. high)	0.61 [0.28, 1.33]	0.81 [0.35, 1.86]
Water flow rate (in ml/min)		**0.57 [0.36, 0.91]**
Temperature (in °C)		1.10 [0.85, 1.42]
Air pressure (in Pa)		0.98 [0.55, 1.77]
CO_2_-value (in ppm)		**2.04 [1.24, 3.37]**
NH_3_-value (in ppm)		1.10 [0.66, 1.83]
Relative humidity (in %)		**0.55 [0.33, 0.94]**
Floor condition (as new vs. worn)		**6.28 [1.89,20.90]**

Within the framework of the final multivariable frequentist model (FM 2), two specifications were considered: Model 1 included explanatory variables at the animal and pen levels, while Model 2 additionally included compartment-level variables. Thus, both models represent FM 2 structures differing only in the number and hierarchical level of predictors. Bold values indicate statistically significant results (*p* < 0.05).

Explanatory variables for the pen level have been considered in model 1. These are the pen size (in $m^{2}$ ), the age of the animals (in days), the stocking density within a pen, animals with skin lesions (“low”- or “medium”-/high-grade lesions in each case in comparison with the reference category no lesions) and the degree of animal contamination within a pen.

Comparing the general outcome of both models show similar estimates at pen-level. Always all factors under study show no statistically significant effect. However, the age of the animals has a statistically significant effect on cough within model 1 and model 2. The estimated odds ratio for age is 1.70 resp. 1.64.

In model 2, compartment-specific variables have been added now. These are the water flow rate (in ml per minute), the measured temperature (in C), the air pressure (in Pa), the carbon dioxide value (CO_2_), the corrected ammonia value (NH_3_) (each in ppm) and the relative humidity (in %), each in one compartment. Also evaluated was the floor condition (as new vs. worn).

Four of these supplemental factors show a statistically significant effect, which again indicates the overarching hierarchical necessity of fitting nested models.

So far, only the fixed effects have been studied. However, both models have also allowed random intercepts for pens and compartments. The values of the estimated variances (VAR), standard deviations (SD), the log-likelihood function and intraclass-correlation (conditional and adjusted), for the comparison of the models are shown in Table 5. Here we notice that the estimated variances and standard deviations for the pen effects are close to zero for all our models. Adding explanatory variables significantly reduces the estimated variance across compartments, suggesting that the distribution of one or more variables varies across compartments.

**Table 5:** Measures for model specificity comparing FM 2 (a random-intercept-only model with pens nested in compartments), Model 1 and Model 2.

	FM 2	Model 1	Model 2
Log-likelihood	−270.97	−262.08	−251.27
Number parameters	3	13	20
Estimated VAR pens	0.20	0.15	0.03
Estimated SD pens	0.45	0.39	0.17
Estimated VAR compartments	2.84	2.92	2.99
Estimated SD compartments	1.68	1.71	1.73
ICC*_c_*	0.43	0.46	0.43
ICC_adj_	0.48	0.48	0.48

The adjusted intraclass-correlation ( $\mathrm{IC}C_{\mathrm{adj}}$ ) considers only the random effects in the model. Here, for model 2, a total of 48% of the variation (compared with the total variation) in the outcome variable cough can be explained by the clustering structure of the data in this model (similar to the FM 2). However, the conditional ${\mathrm{ICC}}_{c}$ (considers fixed and random effects) is slightly higher here at 43%.

### 3.3 Results of Bayesian models

Starting the model selection process from a Bayesian point of view, we ran a non-hierarchical Bayes model with all our explanatory variables. This model claimed that the stocking density and the floor condition in the pens are statistically significant variables for the response.

Since we know of the hierarchical structure of the data, this non-hierarchical model does not account for the pen and compartment effects. Therefore, we ran an intercept only model with varying intercepts for the hierarchical levels pen and compartment. The ICC-value for this model is $\mathrm{IC}C_{\mathrm{adj}}$ = 0.53, meaning that 53% of the variation in the outcome variable can be accounted for by the clustering structure of the data. Splitting this measure into the two hierarchy levels, we get $\mathrm{IC}C_{\mathrm{pen}}$ = 0.03 for the pen level and $\mathrm{IC}C_{\text{compartment}}\;$ = 0.50 for the compartment level, which drives the decision to do not take the pen-level into further consideration.

This leaves us with Bayesian models with random intercepts for the compartment level and all explanatory variables. Within these we accounted for different kinds of (non- and high informative) prior distributions to our models as outlined in Table 6.

**Table 6:** Overview of prior distributions used in Bayesian logistic regression models.

Model	Prior distributions
BM 1 (non-hierarchical)	$\beta_{0}\sim N\;(0,50)$ $\beta_{1},\ldots ,\beta_{15}\sim N\;(0,100)$ - - -
BM 2 (hierarchical) (non-informative)	$\beta_{0}\sim N\;(0,50)$ $\beta_{1},\ldots ,\beta_{15}\sim N\;(0,100)$ $\sigma_{0}\sim \text{InvGamma}\;(0.01,0.01)$ $\sigma_{0i}\sim \text{InvGamma}\;(0.01,0.01)$ $\sigma_{0\mathrm{ij}}\sim \text{InvGamma}\;(0.01,0.01)$
BM 3 (hierarchical) (non-informative)	$\beta_{0}\sim N\;(0,100)$ $\beta_{1},\ldots ,\beta_{15}\sim N\;(0,1000)$ $\sigma_{0}\sim \text{InvGamma}\;(0.01,0.01)$ $\sigma_{0i}\sim \text{InvGamma}\;(0.01,0.01)$ $\sigma_{0\mathrm{ij}}\sim \text{InvGamma}\;(0.01,0.01)$
BM 4 (hierarchical) (high-informative)	$\beta_{0}\sim N\;(0,1)$ $\beta_{1},\ldots ,\beta_{15}\sim N\;(0,10)$ $\sigma_{0}\sim \text{InvGamma}\;(1,1)$ $\sigma_{0i}\sim \text{InvGamma}\;(1,1)$ $\sigma_{0\mathrm{ij}}\sim \text{InvGamma}\;(1,1)$
BM 5 (hierarchical) (high-informative)	$\beta_{0}\sim N\;(0,1)$ $\beta_{1},\ldots ,\beta_{15}\sim N\;(0,1)$ $\sigma_{0}\sim \text{InvGamma}\;(0.5,0.5)$ $\sigma_{0i}\sim \text{InvGamma}\;(0.5,0.5)$ $\sigma_{0\mathrm{ij}}\sim \text{InvGamma}\;(0.5,0.5)$

BM 1: non-hierarchical; BM 2 – BM 3: hierarchical with non-informative priors; BM 4 – BM 5: hierarchical with highly informative priors.

To explicitly incorporate prior knowledge into our hierarchical Bayesian models, we defined informative priors for key parameters based on expert knowledge in the field. The rationale for using informative priors was twofold: first, to stabilize estimation in the presence of limited or noisy data, and second, to restrict the model in biologically plausible parameter spaces. We acknowledge that prior selection can substantially influence posterior inference, particularly in complex hierarchical settings. Therefore, we conducted a sensitivity analysis, demonstrating that the main conclusions of the model remained robust across a range of plausible prior specifications. The improved performance of model BM 5, which used highly informative priors, should thus be interpreted as a consequence of the coherent integration of data and prior information, rather than being solely driven by the prior itself.

The resulting estimated odds ratios and their associated 95% credibility intervals were as follows (Table 7): for stocking density in BM 1 (non-hierarchical), it was estimated at 3.84 with a credibility interval of [2.97, 4.96]. In contrast, BM 2 (hierarchical; non-informative) showed an odds ratio of 9.30 [6.23, 13.89], while BM 3 (hierarchical; non-informative) had an odds ratio of 9.28 [6.19, 13.91]. For BM 4 (hierarchical; highly informative), stocking density yielded an odds ratio of 9.34 [6.15, 14.19], and BM 5 (also hierarchical; highly informative) resulted in an odds ratio of 6.33 [4.35, 9.22].

**Table 7:** Odds ratios with 95% credible intervals from Bayesian logistic regression models.

Factor	BM 1 non-hierarchical	BM 2 hierarchical non-informative	BM 3 hierarchical non-informative	BM 4 hierarchical highly- informative	BM 5 hierarchical highly- informative
Stocking density (in animals/m^2^)	**3.84 [2.97,4.96]**	**9.30 [6.23, 13.89]**	**9.28 [6.19, 13.91]**	**9.347 [6.15, 14.19]**	**6.33 [4.35,9.22]**
Pen size (in m^2^)	0.96 [0.73, 1.27]	1.01 [0.58, 1.76]	1.01 [0.57, 1.76]	1.01 [0.57, 1.76]	0.98 [0.59, 1.63]
age (in days)	1.02 [0.79, 1.32]	1.04 [0.66, 1.61]	1.04 [0.66, 1.62]	1.04 [0.66, 1.63]	1.03 [0.69, 1.56]
Floor condition (as new vs. worn)	**3.84 [2.03, 7.28]**	**7.29 [1.66, 32.93]**	**7.57 [1.79, 34.22]**	**7.46 [1.72 34.69]**	**3.45 [1.16, 10.18]**
Water flow rate (in ml/min)	1.00 [0.76, 1.31]	1.00 [0.58, 1.73]	1.00 [0.57, 1.76]	1.00 [0.57, 1.76]	1.00 [0.60, 1.66]
air pressure (in Pa)	1.00 [0.77, 1.29]	1.00 [0.46, 2.19]	1.00 [0.46, 2.18]	1.00 [0.45, 2.20]	1.02 [0.52, 1.98]
CO_2_-value (in ppm)	1.00 [0.75, 1.33]	1.00 [0.52, 1.92]	1.00 [0.52, 1.92]	1.00 [0.52, 1.93]	1.00 [0.57, 1.76]
NH_3_-value (in ppm)	0.97 [0.77, 1.21]	1.05 [0.52, 2.14]	1.05 [0.51, 2.17]	1.05 [0.50, 2.20]	1.05 [0.56, 1.96]
Temperature (medium vs. high)	2.59 [0.63, 11.43]	3.66 [0.34, 39.97]	3.78 [0.34, 42.82]	3.84 [0.35, 46.82]	1.66 [0.37, 7.42]
Relative humidity (in %)	1.00 [0.75, 1.32]	0.90 [0.44, 1.86]	0.90 [0.44, 1.86]	0.90 [0.43, 1.89]	0.92 [0.49, 1.75]
Skin lesions 1 (none vs. low)	1.22 [0.73, 2.03]	0.98 [0.43, 2.25]	0.98 [0.43, 2.27]	1.02 [0.43, 2.33]	0.97 [0.47, 1.98]
Skin lesions 1 (none vs. high)	0.94 [0.49 1.77]	0.78 [0.29, 2.04]	0.79 [0.30, 2.06]	0.79 [0.30, 2.16]	0.75 [0.33, 1.68]
Animal contamination 1 (none vs. low)	0.86 [0.47, 1.61]	1.16 [0.45, 3.20]	1.19 [0.45, 3.42]	1.21 [0.46, 3.30]	1.24 [0.57, 2.71]
Animal contamination 2 (none vs. high)	0.73 [0.35, 1.55]	0.56 [0.16, 2.07]	0.58 [0.16, 3.42]	0.59 [0.16, 2.07]	0.74 [0.27, 2.09]

BM 1: non-hierarchical; BM 2 – BM 5: hierarchical with random intercepts for compartments (BM 4 – BM 5 use highly informative priors). Credible intervals are based on the posterior distribution and are not directly comparable to frequentist confidence intervals. Bold values indicate statistically significant results (*p* < 0.05).

In our analysis, floor condition emerged as a significant variable influencing the occurrence of cough in pigs. The assessment categorized floor condition into two levels: “new” and “worn.” The data revealed that 3.84 odds ratio (OR) for worn floor conditions indicates that pigs housed in compartments with worn flooring have approximately four times higher odds of exhibiting coughing symptoms compared to those in pens with new flooring.

This finding suggests that the quality of the flooring has a direct impact on respiratory health. Worn or degraded flooring can contribute to increased dust and pathogen exposure, leading to higher instances of respiratory issues among livestock. In this context, it is crucial for farm management practices to prioritize maintaining good floor conditions within pig housing facilities as part of overall biosecurity and animal welfare strategies. The results highlight the importance of addressing environmental factors such as floor condition when evaluating animal health outcomes.

In terms of model specificity measures comparing Bayesian Models (BM 2–5), Table 8 summarizes key statistics including estimated random effects variance ( ${\hat{\sigma }}_{0\;\text{compartments}}$ ) which was estimated at 2.36, 2.37, 2.36, and 2.21 across models BM 2 through BM 5, respectively.

**Table 8:** Model-specific measures from Bayesian models BM 2–BM 5.

	BM 2	BM 3	BM 4	BM 5
${\hat{\sigma }}_{0\;\text{compartments}}$	2.36	2.37	2.36	2.21
ICC_adj_	0.64	0.64	0.66	0.62
ICC_unadj_	0.53	0.54	0.55	0.54

σ^2^₀: estimated variance of the random compartment effect; ICC_adj_: adjusted/conditional intraclass correlation; ICC_unadj_: unadjusted ICC.

**Figure 3:**
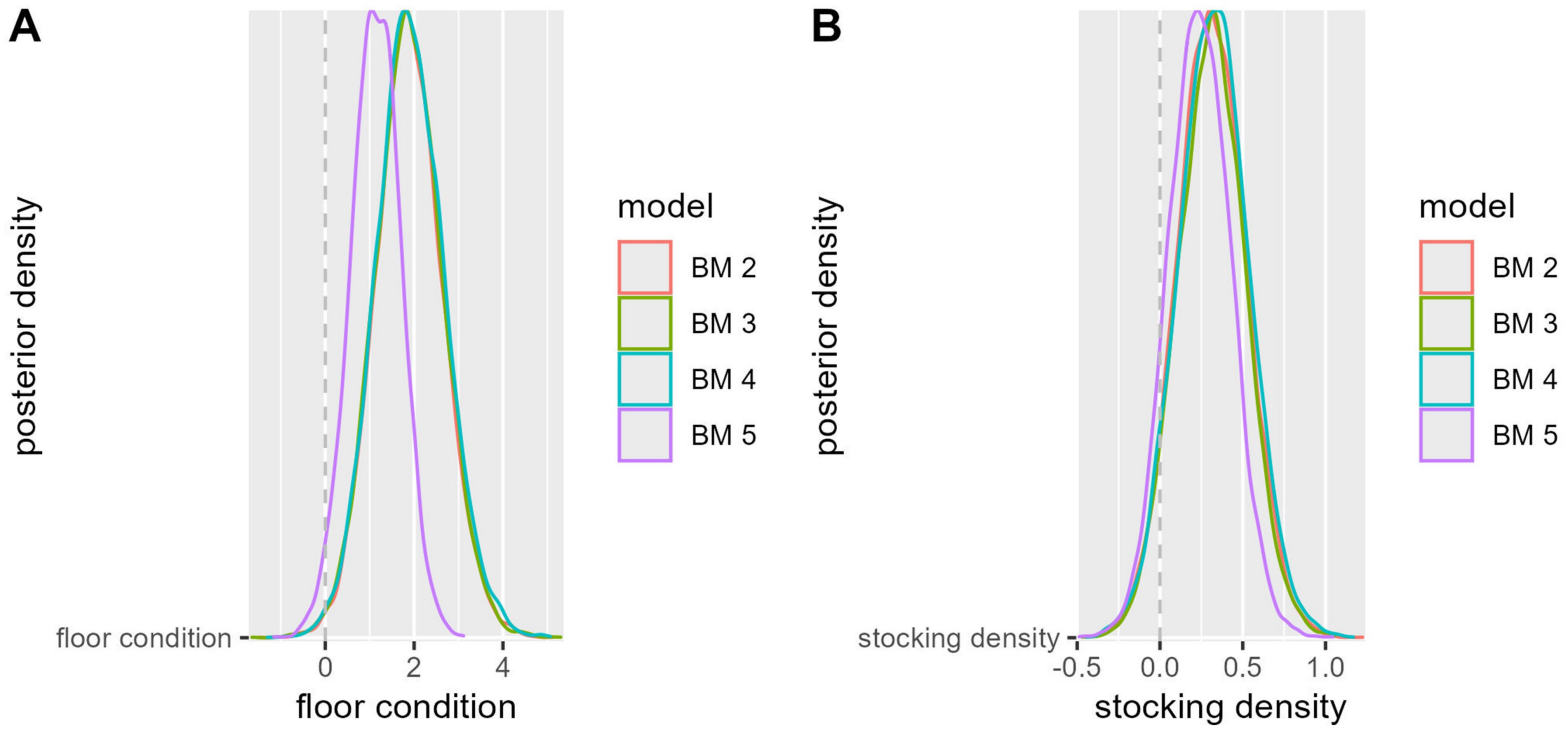
Posterior density distribution for different models (BM 2 – BM 5) in relation to floor condition **(A)** and stocking density **(B)**.

Figure 3A shows the posterior densities for different models (BM 2 – BM 5) in relation to floor condition. Most models are very similar in their density distribution, with the exception of BM 5, which has a higher peak concentration. All models show a main distribution around a positive effect area, which indicates that floor properties have an overall positive influence on the target variable under consideration. The dashed zero reference line marks the boundary between positive and negative, i.e., here preventive effects. None of the models has a substantial density in the negative range, which indicates that restrictions in floor quality always result in increased respiratory problems.

All models (BM 2–BM 5) for the variable “stocking density” (Figure 3B) show similar distributions centered close to zero. This suggests that the overall influence of stock density on the outcome is small or directionless. As the densities cluster around the dashed zero reference line, stocking density appears to play only a minor role for coughing in this collective of farms.

It is noticeable that BM 5 (purple) has a slightly narrower distribution and a greater maximum value than the other models, which could indicate less uncertainty in this estimate. The remaining models (BM 2 – BM 4) show a wider distribution, which could indicate greater uncertainty or variability in the estimate of the effect.

Overall findings indicate that while both hierarchical and non-hierarchical models provided insights into factors affecting cough incidence in pigs, modeling approaches that incorporate random effects offer more robust estimates by accounting for underlying data structures.

### 3.4 Evaluation results

At the beginning, three hierarchical frequentist models (FMs) without explanatory variables were fitted to assess the impact of the data’s clustering structure. The model estimates indicated that, in an “average” pen, pigs have a 35% probability of coughing without accounting for other influencing factors. Subsequently, models incorporating explanatory variables identified significant predictors – namely, stocking density and floor condition – as influential on the incidence of coughing among pigs.

Next, our approach was extended using Bayesian methods. First, all explanatory variables were included in non-hierarchical models, which revealed that stocking density and floor condition significantly influenced coughing incidence. Recognizing the hierarchical structure of the data, intercept-only models with varying intercepts across pen and compartment levels were estimated. These models showed an intra-class correlation (ICC) indicating that 53% of the variation was attributable solely to clustering effects, with the compartment level being dominant. The resulting estimated odds ratios across different Bayesian models highlighted significant variations, particularly with regard to stocking density; inflated estimates were observed when the hierarchical structure was not accounted for, compared to models that did include random effects.

The *loo* package in R was used to perform Leave-One-Out Cross-Validation (LOO), thereby assessing the out-of-sample predictive performance of the hierarchical Bayesian models. Using the loo() function, expected log pointwise predictive density (elpd) scores were computed. The model comparison analysis based on LOO resulted in elpd differences for five Bayesian models. The results are summarized in Table 9.

**Table 9:** Leave-one-out cross-validation results for Bayesian models BM 1 –BM 5.

Model	elpd_diff	se_diff
BM5	0.0	0.0
BM4	−2.8	1.7
BM2	−3.1	1.9
BM3	−3.4	1.8
BM1	−52.0	10.1

elpd_diff: difference in expected log pointwise predictive density compared to the best model (BM5); se_diff: standard error of elpd_diff.

Generally model BM 5 served as the reference with the best predictive performance, while BM 1 showed a substantial drop in performance. Convergence diagnostics were monitored using the Rhat statistic derived from MCMC samples generated with tools like Stan (via the rstan package in R). All chains converged well (Rhat < 1.01), confirming the reliability of the parameter estimates.

Additionally, Bayes R^2^ values were computed to assess the proportion of variance explained by the models. Table 10 summarizes the Bayes R^2^ estimates for various models with and without the inclusion of random effects:

**Table 10:** Bayes R^2^ estimates (posterior median and 95% credible intervals) for models with and without random effects.

Model	Estimate	Est. error	Q2.5	Q97.5
Random effects included				
BIM 1	0.343	0.047	0.242	0.429
BIM 2	0.357	0.041	0.279	0.436
BIM 3	0.363	0.042	0.280	0.441
BM 1	0.137	0.023	0.092	0.181
BM 2	0.428	0.037	0.356	0.502
BM 3	0.432	0.038	0.360	0.507
BM 4	0.444	0.035	0.375	0.511
BM 5	0.424	0.037	0.352	0.496
Random effects not included				
BIM 1	0.000	0.000	0.000	0.000
BIM 2	0.000	0.000	0.000	0.000
BIM 3	0.000	0.000	0.000	0.000
BM 1	0.137	0.023	0.092	0.181
BM 2	0.208	0.031	0.142	0.262
BM 3	0.209	0.031	0.144	0.264
BM 4	0.213	0.031	0.148	0.267
BM 5	0.188	0.031	0.122	0.243

BM 1: non-hierarchical model; BM 2 – BM 5: hierarchical models with random intercepts for compartments (BM 4 – BM 5 with informative priors). BIM 1 – BIM 3: Bayesian intercept-only models with random intercepts for pens (BIM 1), pens and compartments (BIM 2), and pens, compartments, and farms (BIM 3). Credible intervals reflect uncertainty from the posterior and are not equivalent to confidence intervals.

In summary, the Bayes R^2^ results indicate that models including random effects provide significantly higher explained variance compared to models without them, especially evident in the FMs which exhibit no explanatory power without random effects.

## 4 Discussion

The investigation presented here shows an extended version of a model building process for respiratory health in pig production. Therefore, in the discussion section we want to reflect on the implications of our findings in two dimensions by comparing different modeling approaches and their suitability for analyzing complex, hierarchical data first, and by discussing the findings from the viewpoint of veterinary advice to the farmers.

### 4.1 The model selection process and its characteristics

The model selection was based on multiple criteria, including model fit, convergence issues, and the interpretability of results as recommended in Burnham and Anderson (32). Frequentist models offer a straightforward interpretation with clear estimates and confidence intervals, making them widely used in applied research (33). However, they can struggle with complex hierarchical structures and small sample sizes, potentially leading to biased estimates when the assumption of independence is violated (34).

In contrast, Bayesian models integrate prior knowledge into the analysis, allowing for a more understanding of the data and the ability to handle hierarchical structures effectively (35). They provide credible intervals that more accurately reflect uncertainty, especially in small samples or complex models (35). The trade-offs, however, include a need for careful prior selection, which can be subjective and context-dependent (36), as well as increased computational intensity due to iterative simulation methods such as MCMC (37). Hierarchical models, which account for the nested structure of data, further improve precision by modeling variability across different levels (4). While these models yield more stable and realistic estimates, they also introduce additional complexity in both model fitting and interpretation, requiring advanced diagnostics and greater computational resources (11).

The study compared four basic model types, summarized in Table 11.

**Table 11:** Comparison overview of the four underlying models.

Model	Model characteristics				
	Flexibility	Handles clustering	Handles small data	Includes prior knowledge	Complexity
Frequentistic hierarchical	Moderate	Yes	No	No	Low
Bayesian non-hierarchical non-informative	Moderate	No	Yes	No	Moderate
Bayesian hierarchical non-informative	High	Yes	Yes	No	High
Bayesian hierarchical highly informative	Very high	Yes	Yes	Yes	Very high

Convergence issues were observed in models with a high number of explanatory variables, underlining the inherent complexity of hierarchical modeling. Notably, the Bayesian hierarchical model with highly informative priors (BM 5) outperformed its counterparts by delivering the highest predictive accuracy, as evaluated using Leave-One-Out Cross-Validation, and by achieving higher Bayesian R^2^ values that underscored the explained variance. This model provided robust and realistic estimates by appropriately accounting for the hierarchical structure and avoiding the overestimation of effects observed in non-hierarchical models such as BM 1.

The goodness-of-fit measures employed in our analysis – namely the marginal and conditional R^2^, the Intraclass Correlation Coefficient (ICC), and the AIC/AIC_C_ – provided valuable insights into the performance and appropriateness of our models. The marginal R^2^ (R^2^m) quantified the proportion of variance explained solely by the fixed effects, while the conditional R^2^ (R^2^c) captured the overall explanatory power when both fixed and random effects were considered. This distinction underscored the importance of incorporating random effects to account for the hierarchical structure inherent in our data. Similarly, the ICC offered a direct measure of the variability attributable to clustering, highlighting the degree of within-cluster similarity and reinforcing the necessity for hierarchical modeling. Although the explained variance in our models — as reflected in the R^2^ and ICC values ranging from approximately 0.17 to 0.50 — may appear modest, such values are not uncommon in field-based veterinary epidemiological studies. This is largely due to the high biological, environmental, and management-related variability inherent to real-world farm data. The primary goal of our modeling approach was not to fully explain the outcome variable, but rather to detect consistent and meaningful associations between risk factors and respiratory symptoms.

In particular, hierarchical models are designed to capture both fixed effects and random variability between nested levels (e.g., pens, compartments), and much of the unexplained variance may be attributed to unobserved or unmeasurable influences such as transient environmental fluctuations or management decisions, which are not documented. Importantly, several risk factors — such as floor condition and stocking density — emerged as robust predictors across different model structures and prior specifications. Therefore, despite moderate overall model fit metrics, the findings remain highly relevant and applicable in the context of veterinary field epidemiology.

This interpretation is in line with methodological guidance from Gelman and Hill (4) and McElreath (11), who emphasize that low R^2^ values in hierarchical models often reflect natural complexity rather than model inadequacy.

Our results revealed that the Bayesian hierarchical model with highly informative priors (BM 5) demonstrated superior performance compared to its Frequentist counterparts. Notably, BM5 achieved higher conditional R^2^ and ICC values, suggesting that it more effectively captured both the systematic (fixed) and the random variability in the data. In contrast, the Frequentist models, while easier to interpret, tended to produce lower R^2^ estimates and were more prone to inflated effect estimates when clustering was inadequately addressed. Furthermore, model comparison through AIC and AIC_C_ consistently favored the Bayesian approach, albeit with the caveat that its increased computational complexity and sensitivity to prior specification require careful management.

Some of these recommendations support the findings of our analysis, while others offer alternative perspectives that enrich the discussion. For example, Burnham and Anderson (32) emphasize that model selection should be based on multiple criteria – such as parsimony, explanatory power, and convergence behavior – rather than relying solely on fit indices like AIC or BIC. This aligns with our approach of balancing interpretability and model performance. Similarly, McNeish and Stapleton (40) caution against using complex hierarchical models in small-sample contexts without careful consideration, echoing our observation that hierarchical modeling can lead to instability if not adequately supported by the data. Conversely, other studies highlight the value of Bayesian approaches in sparse or nested data scenarios. For instance, Gelman and Hill (4) and McElreath (11) advocate for the use of multilevel Bayesian models, particularly when dealing with complex data structures and uncertainty across levels. These perspectives confirm that there is no one-size-fits-all solution in model selection; instead, the choice depends on the structure of the data, the research questions, and practical considerations such as computational cost and interpretability.

In summary, the integration of these goodness-of-fit measures into our evaluation not only validated our model selection but also highlighted the trade-offs between the Bayesian and Frequentist paradigms. While Bayesian models offer enhanced flexibility and robustness in capturing complex hierarchical structures, they demand rigorous prior selection and greater computational resources. Conversely, Frequentist models provide simplicity and ease of interpretation but may fall short in accurately reflecting the underlying data structure, particularly in the presence of significant clustering effects.

### 4.2 Risk factors for coughing in selected farms with sustainable respiratory problems

Using “coughing in piglets” as the sole response variable has both advantages and disadvantages that should be considered when evaluating its validity and limitations.

On the positive side, coughing is easily observable, making it simple for practitioners to document and collect data quickly. This ease of observation allows for timely identification of potential respiratory health issues, enabling early intervention which is crucial in livestock management where rapid responses are mostly necessary. Additionally, monitoring coughing is cost-effective, as it does not require expensive diagnostic tests or extensive clinical examinations, which can be particularly beneficial for farmers with limited resources.

However, there are significant drawbacks to relying solely on coughing as an indicator. Coughing can arise from multiple factors, including infections, allergies, environmental irritants, or stress, making it challenging to pinpoint the exact cause without additional clinical or even laboratory data. Furthermore, the assessment of coughing can be subjective and may vary among different observers, leading to inconsistencies in data collection and interpretation and introducing an information bias. Coughing is also a non-specific symptom that can be associated with various diseases, complicating the diagnostic process when viewed in isolation. The presence of coughing alone may not accurately reflect the severity of an underlying condition, necessitating consideration of other clinical signs to gain a more comprehensive understanding of the piglets’ respiratory health status.

The analysis of various models revealed that both frequentist and Bayesian approaches were able to identify key factors influencing the occurrence of coughing in pigs, including stocking density, floor condition, and water flow, which are clearly to report and addressed within the farm management process. Specifically, an increase in pen size by one square meter was associated with an odds ratio (OR) of 0.79, suggesting a preventive effect, although this result did not reach statistical significance. In contrast, pigs housed in areas with worn floor experienced more than five times the odds of coughing compared to those on new floor, highlighting the crucial environmental impact as an important surrogate for biosecurity. Additionally, an increased water flow rate demonstrated a protective influence (OR = 0.57), emphasizing the importance of adequate hydration.

These general results are of importance due to the farm population studied here. It should be noted that the VASIB project was not a representative cross-sectional study of German pig production, but rather a highly selected collection of farms with persistent respiratory health problems. It can therefore be assumed that the usual farm management measures and the continuous supervision by the herd veterinarian have already exhausted significant factors for improving animal health. Against this background, it is particularly remarkable that even in this collective, factors still appear to be significant which, from the point of view of animal hygiene and the associated biosecurity measures, can actually already be assumed to be known.

It can be concluded that this may indicate that certain influencing factors are either ignored in agricultural practice or cannot be implemented at all. For example, the factor of floor condition, which is consistently considered to be conspicuous, is a factor that cannot be continuously improved, as this requires structural measures. By taking the hierarchical structure into account, however, there were indications of specific compartments with an increased impact, so that this can ultimately also be understood as an indication for the development of alternative hygiene concepts.

## 5 Conclusion and outlook

The statistical modeling conducted in this study provides valuable insights into predicting clinical outcomes in real-world pig production systems, with a particular focus on respiratory health in piglets. Using both frequentist and Bayesian hierarchical approaches, we identified key risk factors—most notably stocking density, floor condition, and water flow rate—that significantly influence the incidence of coughing. These findings can support veterinarians and farmers in developing targeted management strategies to improve animal health and welfare.

Several limitations must be acknowledged. The reliability of predictions depends strongly on the quality and completeness of the collected data, and missing or misreported information could bias results. Hierarchical models, although powerful, are complex to interpret for non-specialists, and both frequentist and Bayesian approaches rely on assumptions that may not always hold in practice. Moreover, the farms included in the VASIB project were pre-selected due to persistent respiratory health problems, and therefore do not represent the wider population of German pig production systems. This restricts the generalizability of our findings. Bayesian modeling, while advantageous for incorporating prior knowledge and generating predictions for farms outside the dataset, is computationally demanding and may require close collaboration with epidemiologically trained veterinarians for practical implementation.

From a methodological perspective, hierarchical regression models were essential for accurately assessing respiratory health risks. Frequentist methods demonstrated the importance of accounting for clustering effects, while Bayesian approaches refined estimates through the integration of prior information. In particular, the Bayesian hierarchical model with informative priors (BM 5) achieved the highest predictive accuracy, effectively capturing data structure and reliably identifying significant predictors. Nonetheless, model selection outcomes may differ under other data conditions.

From an animal health perspective, the results highlight the importance of optimizing stocking density, maintaining flooring quality, and ensuring adequate environmental conditions as central strategies to reduce respiratory disease in piglet production systems with persistent health challenges.

Looking forward, Bayesian modeling offers a promising avenue for predictive applications in animal health, enabling risk estimation even for farms not included in the study population. By leveraging prior knowledge and explicitly modeling uncertainty, these approaches can guide preventive interventions on a broader scale. Future research should prioritize improving data quality in farm settings, simplifying the communication of complex model outputs for practitioners, and validating results in more diverse and representative farm populations. With these advances, statistical modeling can become an increasingly practical and powerful tool for proactive health management in livestock production.

## Data Availability

The data analyzed in this study is subject to the following licenses/restrictions: the data were collected on an individual basis from farmers and veterinary practitioners. Each participant provided written consent with the understanding that data would not be transferred to a third party. Therefore, any data transfer to interested persons is not allowed without an additional formal contract. Data are available to qualified researchers who sign a contract with the University of Veterinary Medicine Hannover. This contract will include guarantees of the obligation to maintain data confidentiality in accordance with the provisions of the German data protection law. Currently, there is no data access committee or another body who could be contacted for the data. However, for this purpose, a committee will be founded. This future committee will consist of the authors as well as members of the University of Veterinary Medicine Hannover. Interested cooperative partners who are able to sign a contract as described above may contact LK Institute of Biometry, Epidemiology and Information Processing University of Veterinary Medicine, Hannover Bünteweg 2, 30559 Hannover. Requests to access these datasets should be directed to lothar.kreienbrock@tiho-hannover.de.
