# Supplementary material for: Hierarchical modeling of risk factors with and without prior information—the process of regression model evaluation for an example of respiratory diseases in piglet production from daily practice data
Source: Front Vet Sci. 2025 Sep 29;12:1611771. doi: 10.3389/fvets.2025.1611771 (PMC12516706; doi:10.3389/fvets.2025.1611771)
Supplement: Supplementary file 1 [file Data_Sheet_1.PDF]

Tug et al. - Hierarchical modelling of risk factors with and without prior information
– the process of regression model evaluation
for an example of respiratory diseases in piglet production from daily practice data

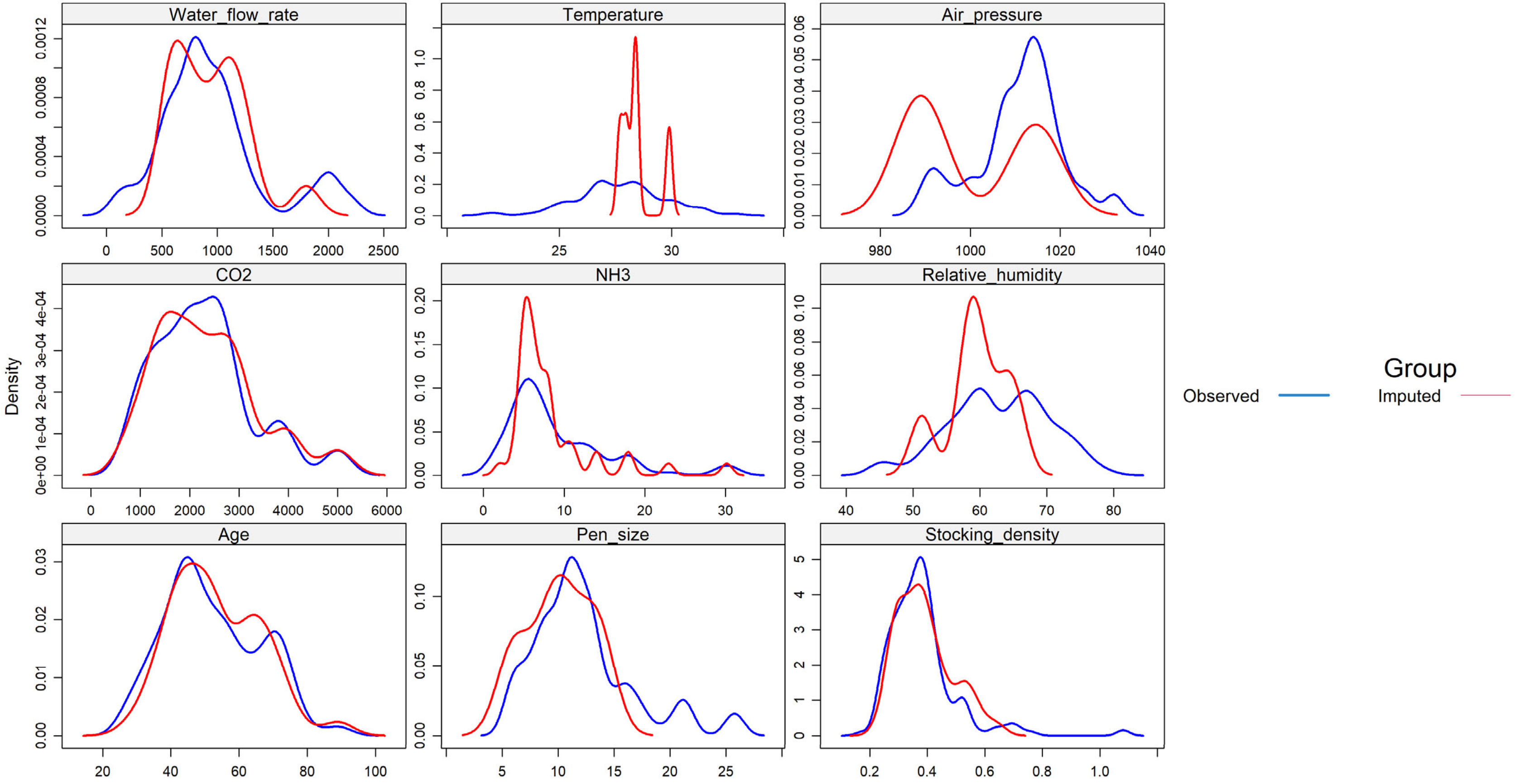

Figure S1: Comparison of density plots for the observed and imputed data for different important variables.

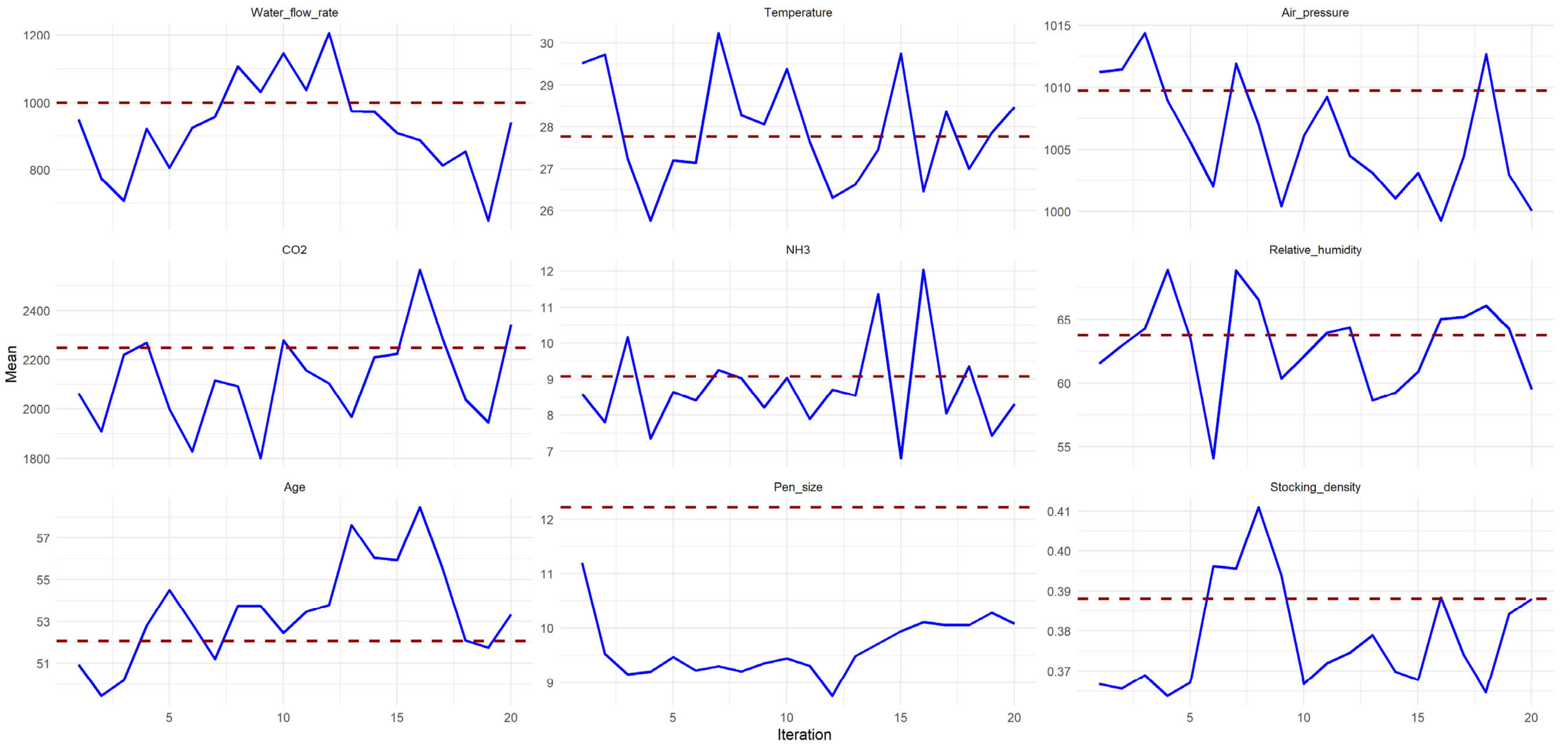

Figure S2: Convergence diagnostics plots different important variables (dashed line observed mean; blue line imputed values over 20 iterations).

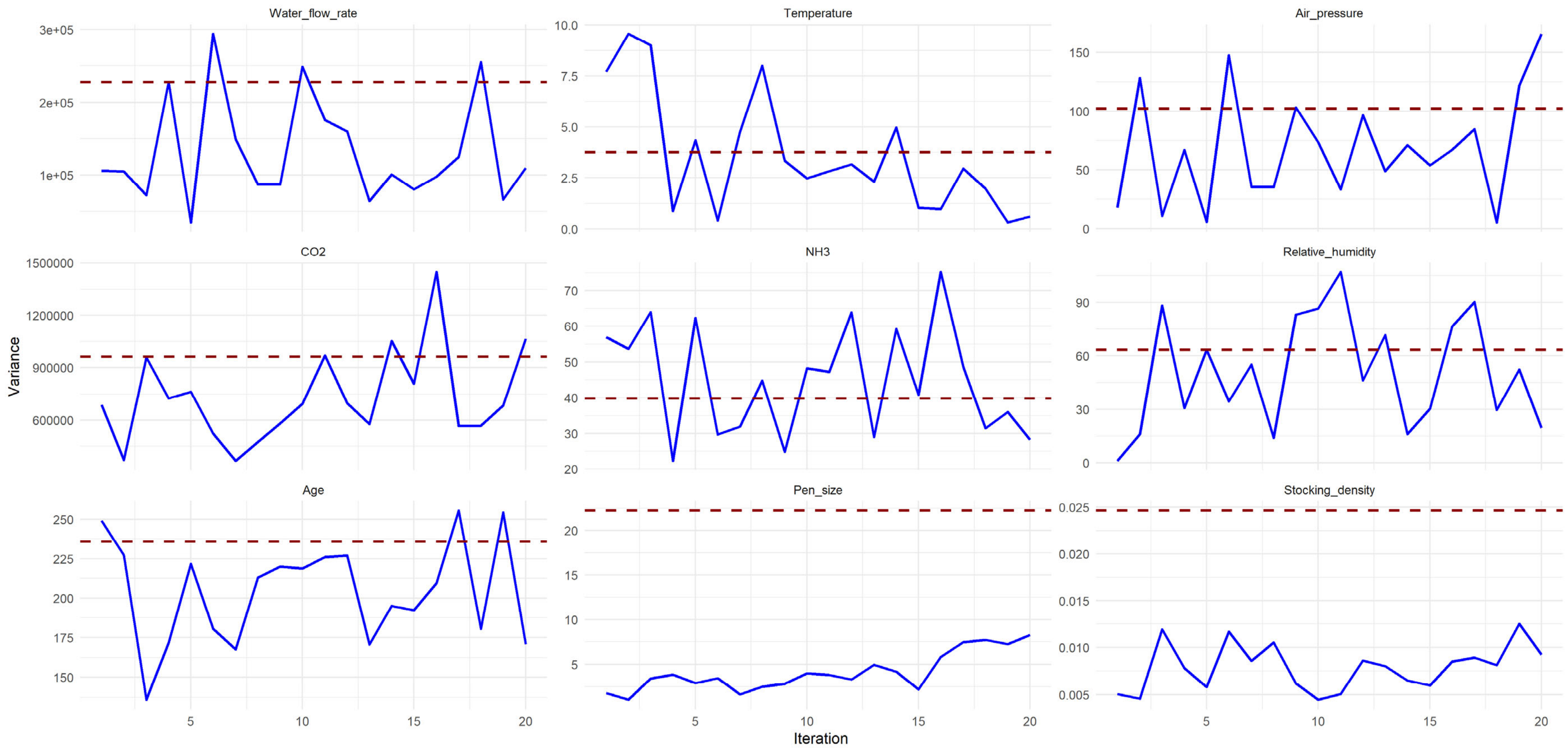

Figure S3: Convergence diagnostics plots different important variables (dashed line observed variance; blue line imputed values over 20 iterations).
